# Redirected Primary Human Chimeric Antigen Receptor Natural Killer Cells As an “Off-the-Shelf Immunotherapy” for Improvement in Cancer Treatment

**DOI:** 10.3389/fimmu.2017.00654

**Published:** 2017-06-09

**Authors:** Olaf Oberschmidt, Stephan Kloess, Ulrike Koehl

**Affiliations:** ^1^Institute of Cellular Therapeutics, Hannover Medical School, Hannover, Germany

**Keywords:** natural killer cells, chimeric antigen receptor, chimeric antigen receptor-associated signaling domain, intracellular chimeric antigen receptor-dependent signaling, cancer immunotherapy

## Abstract

Primary human natural killer (NK) cells recognize and subsequently eliminate virus infected cells, tumor cells, or other aberrant cells. However, cancer cells are able to develop tumor immune escape mechanisms to undermine this immune control. To overcome this obstacle, NK cells can be genetically modified to express chimeric antigen receptors (CARs) in order to improve specific recognition of cancer surface markers (e.g., CD19, CD20, and ErbB2). After target recognition, intracellular CAR domain signaling (CD3ζ, CD28, 4-1BB, and 2B4) leads to activation of PI3K or DNAX proteins (DAP10, DAP12) and finally to enhanced cytotoxicity, proliferation, and/or interferon γ release. This mini-review summarizes both the first preclinical trials with CAR-engineered primary human NK cells and the translational implications for “off-the-shelf immunotherapy” in cancer treatment. Signal transduction in NK cells as well as optimization of CAR signaling will be described, becoming more and more a focal point of interest in addition to redirected T cells. Finally, strategies to overcome off-target effects will be discussed in order to improve future clinical trials and to avoid attacking healthy tissues.

## Introduction

Natural killer (NK) cells are peripheral blood lymphocytes that mediate immune surveillance in regard to virus infected and malignant cells (1–3). For early disease detection and killing NK cells rely on several mechanisms such as inflammatory cytokine secretion [e.g., interferon gamma (IFNγ), tumor necrosis factor alpha (TNF-α), interleukin-10 (IL-10)], receptor ligand binding (e.g., tumor necrosis factor-related apoptosis inducing ligand, Fas ligand) (4), or release of cytoplasmic granule toxins (e.g., perforin, granzyme A, granzyme B, and granulysin) (5, 6) as a result of antibody-dependent cellular cytotoxicity (ADCC) (7).

Recognition of aberrant and stressed cells occurs by means of activating cell surface receptors including natural killer group 2 member D (NKG2D) (CD314), NKp30 (CD337), NKp46 (CD335), and NKp44 (CD336) (8), receptor complex CD94/NKG2C (9), or FCγRIII (CD16) for ADCC (10–12). The counterpart of these activating complexes comprises various inhibitory receptors that usually bind to a variety of different major histocompatibility complex I (MHC I) molecules. Examples for these receptors are several receptors of the killer cell Ig-like receptors (KIRs) family (CD158), NKG2A that pairs with CD94 to a heterodimer (binding the non-classical MHC molecule HLA-E), leukocyte immunoglobulin-like receptor (LILR), natural killer cell receptor protein 1 (CD161), sialic acid-binding immunoglobulin-like lectin-7 (CD328), leukocyte-associated Ig-like receptor 1 (LAIR-1; CD305), killer cell lectin-like receptor G1, carcinoembryonic antigen-related cell adhesion molecule (CD66a), paired immunoglobulin-like receptor α, and CD300a. Each NK cell expresses individually a composition of inhibitory and activating receptors (9). In the resting state, NK cells are in balance receiving signals from activating and inhibitory ligands and no signaling pathway dominates. After adaption to self-MHC I environment, NK cells respond to ligands for activating receptors resulting in killing of malignant cells. Presence of self-MHC I demonstrates inhibitory response. Contrarily, lack of constitutive self-MHC I enhances elimination of aberrant cells (13). At least, NK cell activation by ligand receptor interaction sum up signals received from inhibitory and activation receptors, which cumulates to release perforin and granzymes (cytotoxicity) as well as cytokine production (e.g., IFNγ and TFN-α) mediated by adaptor proteins (DNAX activation proteins DAP10 and DAP12, CD3ζ). These peptides contain immunoreceptor tyrosine-based activation motifs (ITAMs) that become phosphorylated by Src kinase family members and result in at least cytotoxicity and cytokine production.

However, tumors can develop tumor immune escape mechanisms to protect themselves from NK cell attack, e.g., by matrix metalloproteinase-dependent proteolytic cleavage of MHC class I polypeptide-related sequence A and B (MICA and MICB) (14). These soluble immunosuppressive molecules decrease NK cell cytotoxicity by reduction of NKG2D expression that leads to attenuated recognition of target cells. Strategies has been developed to overcome this inhibition using cell modifications such as vector transduction (15) or antibodies bound to the NK cell surface. These bi- and trispecific killer engagers recognize, e.g., CD33 *in vivo* on myelodysplastic syndrome target cells, and induce cell lysis (16). Also, a promising approach is the use of chimeric antigen receptors (CARs) to improve NK cell cytotoxicity. CARs consist of an external recognition domain [single-chain variable fragment (scFv)] combined with a transmembrane domain followed by one or more signaling domains. It has been shown that CARs using CD3ζ and CD28 domains and/or additional 4-1BB (CD137) or 2B4 domains demonstrate an enhanced killing activity (see Table 1).

**Table 1:** Preclinical and clinical investigations of CAR-modified primary human natural killer cells.

	Antigen	Signaling domain	Target cells	Efficacy	Reference or ClinicalTrials.gov identifier
Preclinical studies with cell lines as targets	CD19	4-1BB/CD3ζ	Acute lymphatic leukemia cell lines	+++	(15)
	HER-2	CD28/CD3ζ	Ovarian cancer cell line and breast cancer cell line	+	(17)
	Disialoganglioside 2 (GD2)	2B4/CD3ζ	Neuroblastoma cell line	+++	(18)
	CD19	2B4/CD3ζ	ALL cell lines	+++	(18)
	CD19	4-1BB/CD3ζ	B-ALL cell line	+++	(19)
	CD19	4-1BB/CD3ζ	B-ALL cell lines and B cell lymphoma cell lines	++ to +++	(20)
	Natural killer group 2 member D ligands	DAP10/CD3ζ	ALL cell lines and several solid tumor cell lines	+ to +++	(21)
	HER-2	CD28/CD3ζ	HER-2-expressing cell lines	n.a.	(22)
	CD19	4-1BB/CD3ζ	B-ALL cell lines	+	(23)
	CS1	CD28/CD3ζ	Myeloma cell lines	Data not shown	(24)
	CD20	4-1BB/CD3ζ	CD20^+^ B-cell non-Hodgkin lymphoma cell lines	++ to +++	(25)
	Epidermal growth factor receptor (EGFR)	CD28/CD3ζ	Glioblastoma cell lines	+	(26)
	Prostate stem cell antigen (PSCA)	DAP12	several PSCA^+^ tumor cells	(+) to +++	(27)
	CD19	CD28/4-1BB/CD3ζ	CD19^+^ leukemia cell line	+ to +++	(28)
	EGFR	CD28/CD3ζ	Breast cancer cell lines	+	(29)
	GD2	CD28/4-1BB/CD3ζ	Ewing sarcoma cell lines	+ to ++	(30)

Preclinical studies with patient malignant cells as targets	CD19	4-1BB/CD3ζ	Acute lymphatic leukemia	+++	(15)
	CD19	2B4/CD3ζ	Acute lymphatic leukemia	+++	(18)
	CD19	4-1BB/CD3ζ	B-CLL cells	+++	(19)
	CD19	4-1BB/CD3ζ	B-ALL cells	+	(23)
	EGFR	CD28/CD3ζ	Glioblastoma stem cells	(+)	(26)

Clinical trials	CD19	4-1BB/CD3ζ	B-lineage acute lymphoblastic leukemia	n.a.	NCT 00995137
	CD19	4-1BB/CD3ζ	B-lineage acute lymphoblastic leukemia	n.a.	NCT 01974479
	CD19	CD28/CD3ζ	B-lymphoid malignancies	n.a.	NCT 03056339

*(+), cytotoxicity <25%; +, cytotoxicity 25–49%, ++, cytotoxicity 50–75%, +++, cytotoxicity >75%*.

Most published preclinical and clinical studies with CAR-modified immune cells comprise T cells. On the NK cell side, publications are mainly restricted to NK cell lines as reviewed in Ref. (31, 32). Less is known about CAR-engineered primary human NK cells as alternative effector cells since the advantages of NK cells are the limited lifespan of several weeks or months (2, 33) and the absent formation of memory cells that persist in patients as observed in CAR T cells. That means multiple dose of CAR NK cells might be safely administered to patients. The present review will discuss the use of primary NK cells isolated from peripheral blood for CAR engineering.

## Signal Transduction in NK Cells

There is a competitive equilibrium between different opposing pathways (13, 34) that culminate at least in activation or inhibition of NK cells depending on the cell surface complexes that are formed by non-covalent associations between distinct transmembrane ligand-binding and signaling adaptor proteins. The Src (sarcoma) family kinases seem to be essential in these interactions because the enzymes are involved in receptor clustering in these microdomains that may facilitate receptor phosphorylation (35, 36).

Starting with NK-cell–target-cell interactions on the surface, this leads to induction of signaling pathways and at least to release of cytotoxic granules (e.g., perforin, granzyme A/B, and granulysine) and/or secretion of cytokines (e.g., IFNγ and TNF-α).

### Activation Receptors

Natural cytotoxicity receptors (NCRs) as NKp30 and NKp46 can couple to CD3ζ that contains several ITAMs (37). NKp44 instead associates with the ITAM-bearing adaptor DAP12. In the next step, tyrosine residues of the ITAM sequences are phosphorylated by protein tyrosine kinases of the Src family. This leads to recruitment of protein tyrosine kinases of the Syk family (e.g., Syk or ZAP70; spleen-associated tyrosine kinase or zeta-chain-associated protein kinase 70) and transmembrane adaptor molecules (e.g., linker for activation of T cells and non-T cell activation linker) that provide multiple docking sites for Syk family kinases. These associations of different signaling partners initialize activation and phosphorylation of multiple partners of signaling pathways such as PI3K (phosphatidylinositol-4,5-bisphosphate 3-kinase) or members of Vav family resulting in release of lytic granules and leading to cytotoxicity (9). The activation of a single NCR seems to start an activation cascade in which different NCRs cross talk to each another for amplifying activating signals (e.g., cross talk between NKp30, NKp44, and NKp46) (38).

Natural killer group 2 member D (CD314) is non-covalent associated with transmembrane adaptor protein DAP10. This pathway is independent of Syk family tyrosine protein kinases (39) and involved PI3K in its signaling cascade. After ligand binding (MICA, MICB, or divers UL16-binding proteins), phosphorylation of a tyrosine-based DAP10 motif by Src family kinases creates binding sites for p85 subunit of PI3K or for the adaptor protein complex Grb2–Vav1 (growth factor receptor-bound protein 2-vav guanine nucleotide exchange factor 1). The result is exocytosis of lytic granules (e.g., perforin, granzyme A/B, and CD107a) in response to PLC (phospholipase C)-γ2-induction (40–43).

For NK cell activation, Vav proteins are essential. Depending of the NCR and of the DNAX proteins, different Vav proteins are involved, e.g., Vav1 is part of the signaling with NKG2D/DAP10 (39–41), whereas Vav2 and Vav3 take part of the DAP12 signaling cascade (44). Vav proteins are involved in a GTPase-dependent reorganization of the cytoskeleton to mediate the directed release of the granules (9).

The NK cell-activating receptor CD226 (DNAX accessory molecule 1) lacks any ITAM. Instead, intracellular signaling starts with phosphorylation of a serine and a tyrosine residue by protein kinase C. This step is critical for association of CD226 to lymphocyte function-associated antigen 1 at the cell surface and facilitates simultaneously cytoplasmic signaling involving Src kinase, Vav1, and PLC-γ2 leading to NK cell activation (45).

The receptor 2B4 has been characterized as costimulatory for activation receptors (e.g., for CD226). A complex formed of CD226 and 2B4 triggers NK cell degranulation, activates PLC-γ2, and increases Ca^2+^ intracellular flux (46). On one hand, 2B4 is sufficient to induce IFNγ release alone (47), on the other hand, 2B4 demonstrated enhanced cytokine secretion after cross linking with NKG2D (48).

### Inhibitory Receptors

After binding of MHC class I molecules to inhibitory receptors, the inhibitory signaling cascade starts with phosphorylation of one or more ITIM sequences (immunoreceptor tyrosine-based inhibitory motif). Detailed mechanism of phosphorylation is unknown but tyrosine kinases that are involved in activation pathways have been expected. After tyrosine phosphorylation, the phosphatases Src homology 2 domain-containing protein tyrosine phosphatase 1 (SHP1) and SHP2 bind to ITIMs (49) and subsequently recruit additional molecules such as inhibitory C-terminal Src kinase Crk (for LILR and LAIR-1) or β-arrestin 2 (for KIRs). ITIM-bound SHP starts to dephosphorylize specifically Vav1 or other pivotal proteins to inhibit clustering of receptors and cytoskeleton rearrangements (50).

Killer cell Ig-like receptor and CD94/NKG2A initiate an alternative signaling pathway that also results in inhibition. The binding of the tyrosine kinase c-Abl and the subsequent phosphorylation of adaptor protein Crk (CT10 regulator of kinase) by c-Abl cause dissociation of Crk from protein complexes that are involved in NK cell activation (51, 52). Inhibition of NK cells is achieved and lysis of target cells decreased.

A second MHC class I independent pathway is composed of the inhibitory receptor T cell immunoglobulin and ITIM domain (TIGIT) and the putative weak-activating receptor CD96. For inhibitory signaling of TIGIT, the intracellular motifs immunoglobulin tail tyrosine and/or ITIM are phosphorylated after ligand binding following recruitment of SHP1 and Grb2 that result in blocking the pathways of PI3K and mitogen-activated protein kinase. CD96 contains a cytoplasmic ITIM as well as a YXXM motif that is a putative binding sites for the p85 subunit of PI3K that may lead to NK cell activation. Both receptors, TIGIT and CD96, were found to counterbalance the costimulatory receptor CD226 and limit NK cell-mediated cytotoxicity and IFNγ release (46, 53).

## Optimization of CAR Signaling in NK Cells

**Figure 1:**
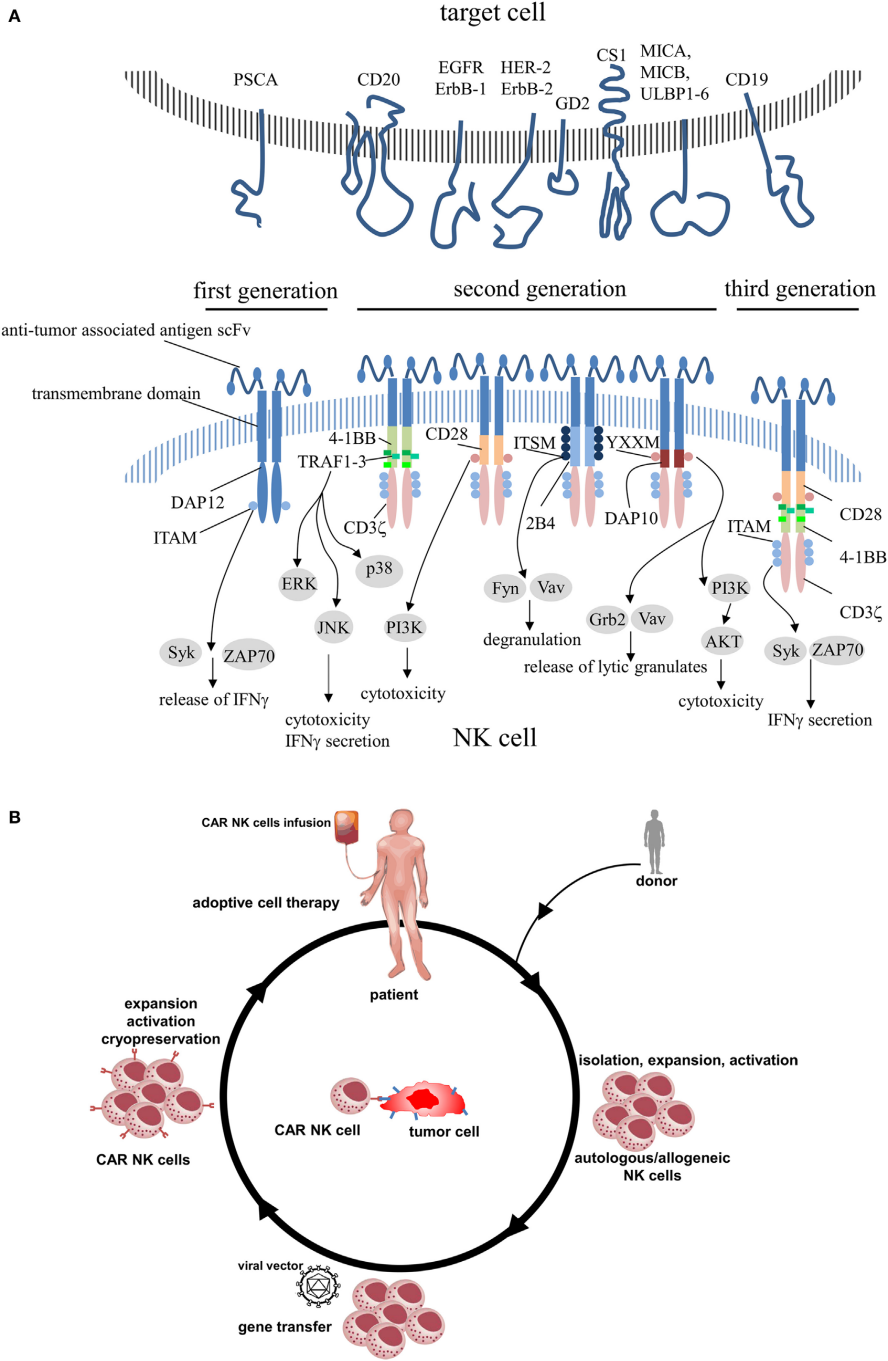
**(A)** Schematic structures of various chimeric antigen receptors applied in engineered primary human NK cells including its intracellular signaling domains. **(B)** CAR NK cell therapy. Autologous NK cells or donor NK cells (allogeneic) are isolated, expanded, and activated by cytokines. After modification of NK cells to express CAR, NK cells are expanded, activated, and administered to the patient or frozen for long-term preservation. PSCA, prostate stem cell antigen; EGFR, epidermal growth factor receptor; HER-2, human epidermal growth factor receptor 2; GD2, disialoganglioside 2; CS1, CD2 subset 1; MICA/B, MHC class I polypeptide-related sequence A/B; ULBP1-6, UL16-binding proteins 1–6; DAP, DNAX-activation protein; ITAM, immunoreceptor tyrosine-based activation motif; Syk, spleen-associated tyrosine kinase; ZAP70, zeta-chain-associated protein kinase 70; TRAF, tumor necrosis factor receptor-associated factor; ERK, extracellular signal-regulated kinase; JNK, c-Jun N-terminal kinase; I3K, phosphatidylinositol-4,5-bisphosphate 3-kinase; ITSM, immunoreceptor tyrosine-based switch motif; Fyn, Src family tyrosine kinase; Vav, vav guanine nucleotide exchange factor; YXXM, phosphorylation motif; Grb2, growth factor receptor-bound protein 2; AKT, protein kinase B.

Chimeric antigen receptors contain an extracellular region of a scFv that was fused to transmembrane domain and cytoplasmic signaling components. The antibody-derived scFv domain is involved in antigen recognition and immune synapse formation, whereas the endodomains are responsible for cell activation. Because CAR constructs are premised on a modular system, it is feasible to combine any scFv with any signaling or cosignaling domain. First-generation CARs that included only signaling motifs derived from CD3 (ζ or γ chain) (13, 54) were fully capable to activate murine CTL hybridoma cells (55), although no additional intracellular signaling region was added. But some tumors were able to inactivate CAR-engineered cells and leading them to anergy (54). To prevent this effect and to improve CAR functionality, subsequent CAR designs incorporated additional costimulatory domains (CD28, 4-1BB, OX40, and 2B4) and evolved to CARs of the second- (addition of one costimulatory domain) or third-generation (addition of more than one costimulatory domain) (Figure 1A).

The motif CD28 is most common in these CAR constructs but is not naturally expressed in human NK cells (56). In T cells, the mode of action of this costimulatory molecule starts with phosphorylation of its intracellular tyrosine residues by PI3K following recruitment of Grb2 and results in activation of protein kinase B (PKB/Akt) and in IL-2 production (57). The advantage for NK cells is still in discussion although for T cells CD28 demonstrates high effectivity (58).

4-1BB is a surface protein discovered on activated T cells (59) that is often used in CAR constructs for NK cells (28). For domain 4-1BB (CD137), costimulation could be clearly detected in T cells (60), but there are conflicting data for NK cells. Navabi et al. demonstrated neither improved NK cell cytotoxicity nor enhanced IFNγ production (61) after NK cell stimulation by 4-1BB ligands in contrast to augmented NK cell-killing capacity as reported in Ref. (62, 63).

The transmembrane adaptor polypeptide DAP10 is originally associated with NKG2D. Comparing the CAR constructs anti-CD19-DAP10 and anti-CD19-CD3ζ, both CARs evoke NK cell cytotoxicity but anti-CD19-CD3ζ exhibited higher antitumor activity than anti-CD19-DAP10 molecules (15). The combination of both signaling domains DAP10 and CD3ζ resulted in secretion of several cytokines (e.g., IFNγ and TNF-α) as well as in a vast release of cytotoxic granules that both increased NK cell cytotoxicity (21).

DAP12 is involved in signal transduction of activated NK cells and is associated with activating receptors such as NKG2C or NKp44. Transmission of intracellular signaling occurs *via* a single ITAM compared to CD3ζ containing three ITAMs (9). Therefore, DAP12 provides an alternative signaling pathway resulting in antitumor activity of NK cells. First investigations assessed DAP12-based CARs in NK cell line YTS (64) as well as in primary human NK cells (27). Combinations of scFv against prostate stem cell antigen (PSCA) with DAP12 exhibit an improved cytotoxicity and increased IFNγ release in primary NK cells compared to CAR NK cells expressing the first-generation CD3ζ-based construct anti-PSCA-CD3ζ (53). This concept without CD3ζ-signaling domain may promise new opportunities to redirect NK cells to resistant target cells.

2B4 (CD244) is a member of the signaling lymphocytic activation molecule family and contains four immunoreceptor tyrosine-based switch motifs (ITSMs) of which the first and second is associated with activation of stimulatory pathways in NK cells (65). Altvater et al. (18) investigated the signaling component 2B4 combined with CD3ζ in primary human NK cells and compared this CAR construct with CAR molecules incorporated either 2B4 or CD3ζ signaling element. As a result, induction of cytokine secretion failed when 2B4 is the sole signaling compound in CAR molecules.

Instead, combination of the domains 2B4 and CD3ζ demonstrated enhanced cytokine secretion (IFNγ and TNF-α) and release of cytolytic granules. In addition, comparable results were observed for a 4-1BB-CD3ζ CAR construct demonstrating equality of 2B4 and 4-1BB signaling domains in combination with CD3ζ.

Similar to 4-1BB, OX40 (CD134) is a TNF receptor on the surface of lymphatic cells (e.g., T cells, NK cells, and NK-like T cells) (66). This costimulatory molecule is involved in recruitment of TNF receptor-associated factor adaptor proteins and leads to cell survival and cytokine release (67, 68). OX40 is often part of third-generation CARs in T cells that show improved signaling capacities based on putative upregulation of PI3K pathway and lead to enhanced cytokine production and cytotoxicity (69), but was not integrated yet in CAR constructs neither for NK cell lines nor for primary NK cells. Because of its costimulatory potential, OX40 may present a promising candidate for improved endogenous CAR signaling in NK cells.

## Preclinical Investigations with Primary Human CAR NK Cells

To date, several preclinical studies have been investigated primary human CAR-modified NK cells directed against various antigens (Table 1). However, compared to CAR T cells that already entered clinical studies, there is only a small number of clinical investigations using CAR NK cells (Table 1).

Most preclinical data describe primary human CAR NK cells directed against CD19 and few against CD20, human epidermal growth factor receptor 2, disialoganglioside 2, epidermal growth factor receptor, and PSCA (references see Table 1).

Mostly, second-generation CARs use CD3ζ in combination with 4-1BB, DAP10, or 2B4, respectively, and result in strong efficacy based on upregulation of the PI3K/AKT pathway. By contrast, CD3ζ constructs with CD28 led to less cytotoxicity. High efficacy could also be revealed by third-generation CARs (CD28/4-1BB/CD3ζ) (28, 30) and a DAP12-based first-generation CAR (27). There is a long-standing discussion that costimulatory domain combines best to CD3ζ. For CAR T cells, investigations suggest that constructs containing 4-1BB may be superior (70), but this has not been yet evaluated for CAR NK cells. In addition, so far safety aspects have not been addressed extensively in CAR NK cells and are under discussion.

Although feasibility and efficacy could be shown for all mentioned constructs in Table 1, safety aspects have to be clarified in detail in an ongoing discussion.

First clinical studies followed the success of CAR T cell trials redirecting NK cells against CD19. These antiCD19-4-1BB-CD3ζ CAR NK cells were administered to patients with B-ALL (NCT 00995137; NCT 01974479) but results have not been published to date. The first study comprises expansion of donor-derived NK cells cocultured with irradiated and gene-modified K562 cells that expressed surface bound IL-15 and 4-1BB 1. The second trial expands IL-2-activated haploidentical NK cells before administering to pediatric and adult patients. Recently, a third study (NCT 03056339) started for patients suffering from relapsed and/or refractory B-cell lymphoma or leukemia. Genetically engineered NK cells derive from umbilical cord blood (CB) and express antiCD19-CD28-CD3ζ CAR, the iCasp9 safety switch as well as IL-15.

## Off-The-Shelf (OTS) Implications for Cancer Treatment

Antigen specificity of CAR NK cells is independent of the recipient’s human leukocyte antigen (HLA) type. This feature is the prerequisite for targeting the same antigen on several tumor types even if recipients demonstrate a high variability of HLA. There may be no need any more to customize individual therapies for each patient. Implementation of a cell bank with cryopreserved immune cells that are allogeneic and genetically modified may solve availability and reduce cost of treatment. Developing OTS therapies means that portions of immune cells will be manufactured (and modified) in advance, stored in cryopreservation, and infused on demand as required by attending physicians (Figure 1B). It has been shown in several studies that administration of haploidentical NK cells to patients with relapsed acute myelogenous leukemia cause good clinical effects without graft versus host disease (GvHD) as reviewed in Ref. (71). For this reason, it seems to be a successful strategy to set a strong focus on CAR NK cell-based immunotherapies (see Table 1), although Shah et al. recently observed GvHD after infusion of *ex vivo* expanded activated allogeneic NK cells (72).

The ideal source for CAR NK cells as OTS products is still in discussion. The cell line NK92 has been described as an option that can be easily transduced and irradiated before administration (73, 74). On the other hand, umbilical CB is well known to be a good source for primary NK cells (75, 76). But limitations as immature phenotype or restriction of NK cell amount should be kept in mind (77, 78), which might be circumvent by refined protocols for primary NK cell *ex vivo* expansion and activation (79) especially in regard to GMP compliance.

A subset of NK cells has been described in mouse and human that demonstrated long-lived capacity for several months (80). These “memory-like” NK cells respond to antigens in second confrontation and show enhanced effector function and expansion. They even may prevent leukemia relapse by a robust cytokine production (81, 82) and may therefore be beneficial in general in long-term antitumor responses. For safety reason, a CAR suicide system should be integrated in CAR NK cells to limit circulation of CAR effector cells in patients (83) and to restrict putative toxic side effects as demonstrated for CAR T cells (84, 85).

## Strategies to Overcome Off-Target Toxicities

The choice of tumor antigens that can be recognized by CARs depends on the unique and selective character of the antigen for target cancer cells. These regular antigens mainly show increased expression on tumor tissues but are also detectable on normal tissues, often in a minute amount. For this reason, on-target toxicities may appear in clinical studies that have been described for CAR T cells (86–88). On the other hand, off-target toxicity attacks tissues and organs that do not express the antigen but CAR constructs can bind unspecifically. For primary human CAR NK cells, toxicity reports have not been published yet but recognition of specific tumor targets are the base for safe and effective CAR constructs.

To increase selectivity of CAR molecules and reduce putative off-target effects, different strategies have been developed, e.g., combination of two extracellular domains in a tandem structure (89, 90) or of two independent constructs to form bispecific CAR molecules (91). A second concept describes CAR constructs that triggers the release of pro-inflammatory IL-12. The composition of CAR resulted in expression of IL-12 after antigen binding to the extracellular CAR domain (92). Recently, Wu et al. developed a split CAR construct that needs a dimerizing small molecule to form a functional unit. This new strategy promises control of timing, location, and dosage of CAR activity and thereby a possible mitigation of toxicities (93). A similar concept demonstrates the use of an inducible molecular switch off (94). When exposed to a dimerizing drug, the fusion protein iCasp9 is activated and triggers apoptosis in all gene-modified cells. In general, all strategies have been shown for engineered CAR T cells, except the last one that has also been evaluated in the murine model using modified NK cells (95) and even for primary CB-derived NK cells expressing antiCD19 CAR molecules [unpublished data mentioned in Ref. (96)].

## Conclusion

In the next years, the possibility of unlimited access to cryopreserved NK cells from CB or third party donors may revolutionize therapy options for cancer patients. Although discussions about best source of NK cells and the question of long-living NK cells have not been finished yet, generation of redirected NK cells against new targets is in rapid progress. Demonstrated results using CAR technologies are auspiciously and may improve cancer therapy also by implemented novel safety strategies. Furthermore, combined immunotherapies using checkpoint blockade monoclonal antibodies to overcome inhibitory signals (e.g., anti-KIR or anti-TIGIT) may enhance CAR NK cell activity.

## Author Contributions

OO performed the review of the literature and wrote the manuscript. SK and UK edited the manuscript.

## Conflict of Interest Statement

The authors declare that the research was conducted in the absence of any commercial or financial relationships that could be construed as a potential conflict of interest.
